# Radiation holidays stimulate tumor immunity

**DOI:** 10.18632/oncotarget.4608

**Published:** 2015-06-23

**Authors:** Laura Surace, Matthias Guckenberger, Maries van den Broek

**Affiliations:** Institute of Experimental Immunology, University of Zurich, Zurich, Switzerland

Radiotherapy is a standard treatment for cancer and is used since a long time as a stand-alone therapy or in combination with surgery and systemic therapies including immunotherapy. Its acts through induction of irreversible DNA damage to which tumor cells are more susceptible because of frequent mutations in DNA repair pathways.

Fractioned radiotherapy, given as daily low doses during multiple weeks, was established as standard protocol in the clinics because it allows recovery of normal tissues. Recently, new technologies were developed that more precisely target radiation to the tumor enabling the delivery of high doses in fewer fractions (hypofractionated or stereotactic radiotherapy) [1].

Recent data suggest that radiotherapy promotes an inflammatory response in the tumor, which supports tumor-specific immunity and actually, efficacy in pre-clinical models seems to depend on concomitant immune stimulation [2-4].

Inflammation is a useful response to disturbance including infection and tissue damage and is instrumental in clearing pathogens and necrotic cells as well as in tissue repair. Moreover, inflammation provides essential innate stimuli to the subsequent activation of protective adaptive immunity. Because inflammation is a potent and destructive response, it must be tightly regulated and resolve as soon as the trigger is eliminated.

Acute inflammation is a relatively short and self-limiting process that culminates in the recruitment and activation of immune cells to the site of action through the production of growth factors, cytokines and chemokines. When the trigger is gone, the production of anti-inflammatory cells and factors terminates this process. In case of a persisting stimulus, which can be an infectious agent, cancer or chronic disturbance, the inflammatory reaction is not resolved but becomes chronic. In order to limit excessive tissue damage, the nature of the inflammatory responses changes under chronic conditions and displays features of simultaneous tissue destruction and repair, angiogenesis and immunosuppression.

Chronic inflammation was correlated with cancer in 1858 by Virchow and is now considered an established hallmark for cancer [5]. Paradoxically, Coley reported in 1893 injection of heat-killed bacteria (Coley's toxin) resulted in tumor regression. This apparent contradiction may be explained by the nature of the inflammation: Chronic inflammation is tumor-promoting, whereas acute inflammation supports protective anti-tumor immunity [6].

Along this line, we showed in mice with established, syngeneic tumors that a single dose of 20 Gy controls tumor progression and promotes a local, transient activation of complement, a potent pro-inflammatory pathway. This resulted in local production of anaphylatoxins (C3a and C5a) that proved crucial to the stimulation of tumor-specific immunity and therapeutic efficacy [3]. When we treated mice with established tumors with fractions of 1.5 or 7 Gy delivered on 5 consecutive days tumor progression was inhibited as well, but neither increased infiltration by leukocytes, specifically CD8+ T cells, nor protective effector function of such T cells was observed in chronically irradiated tumors [3]. In addition, our data show that each dose induces complement activation, which results in a state of chronic activation if radiation is given over a prolonged time on consecutive days. The lack of increased infiltration by leukocytes and specifically of CD8^+^ T cells on the one hand and increased infiltration by regulatory T cells on the other hand after 5 irradiations can be explained by radiosensitivity of effector T cells that were recruited after the first dose and by immunosuppressive chronic inflammation resulting from multiple irradiations. These observations explain the apparently conflicting data by Elvington showing that inhibition of C3 activation improves the efficacy of fractionated radiotherapy [7].

Taken together, we propose that daily radiotherapy results in a chronic inflammatory phenotype that does not promote immune effector but rather regulatory T cells, whereas a single dose and presumably also to repeated doses given with yet to be determined interval results in repeated peaks of immune stimulating, acute inflammation.

**Figure 1:**
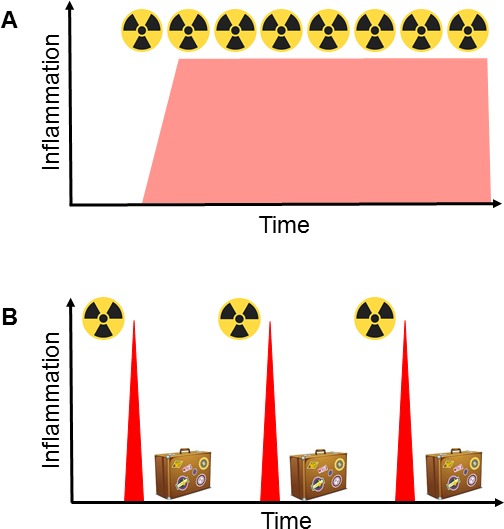
Avoiding chronic inflammation through the use of hypo-fractionated radiotherapy with radiation holidays (A) Standard hyper-fractionated radiotherapy is given as daily fractions of 1.5-2 Gy during a prolonged period. This protocol results in chronic inflammation, which is immunosuppressive, supports angiogenesis and is a hallmark of progressive cancer. (B) We propose to give hypo-fractionated radiotherapy as isolated high-dose fractions with radiation holidays between the treatments. This protocol results in repeated peaks of acute inflammation, which stimulate protective immunity.

Although many aspects are still unknown and require further investigation, we speculate that irradiation with few radiotherapy fractions of higher dose and with a break between the fractions may result in superior therapeutic responses compared to daily treatments (Figure 1).
